# Gender Disparities Among Academic Vitreoretinal Specialists in the United States With Regard to Scholarly Impact and Academic Rank

**DOI:** 10.7759/cureus.39936

**Published:** 2023-06-04

**Authors:** Deniz Oncel, Sapna Syal, Damla Oncel, Nelson A Reyes, Banu Acikalin

**Affiliations:** 1 Department of Ophthalmology, Stritch School of Medicine - Loyola University Chicago, Chicago, USA; 2 Department of Ophthalmology, State University of New York Downstate Medical Center, Brooklyn, USA; 3 Department of Ophthalmology, Rutgers University, Piscataway, USA; 4 Department of Ophthalmology, Fatih Sultan Mehmet Training and Research Hospital, University of Health Sciences, Istanbul, TUR

**Keywords:** gender disparities, retina, vitreo retina, ophthalmology, h-index, retina specialist, gender disparity, gender

## Abstract

Background and objective

While men outnumber women in the specialty of ophthalmology in general, the subspecialty of vitreoretinal surgery in particular has the highest percentage of men across all ophthalmic subspecialties. This study aimed to analyze the gender disparities regarding the publication productivity and academic rank of academic vitreoretinal specialists in the United States (US).

Methods

This cross-sectional study evaluated 116 ophthalmology residency programs in the US participating in the 2022 San Francisco Match. The academic vitreoretinal faculty from each ophthalmology residency program was included. The information on gender, academic rank, and publication activity in terms of the h-index were collected from institutional websites, the Scopus database, and the National Library of Medicine PubMed website.

Results

A total of 467 academic vitreoretinal specialists were identified. Among them, 345 (73.9%) were men, and 122 (26.1%) were women (p<0.001). When the academic ranks were analyzed, a higher number of men (43.8%) were found to hold the rank of full professor as compared to women. Furthermore, a higher number of women (47.5%) were found to hold the rank of assistant professor as compared to their male colleagues. Regarding the number of publications, in all academic rank categories, women had a significantly lower number of publications compared to men (p<0.001). Men also had a higher publication productivity or scholarly impact [h-index=15.2 ± 0.82 standard error of the mean (SEM)] compared to women (h-index=12.8 ± 0.99 SEM) (p=0.0004). Higher h-index correlated with higher academic rank, from assistant professor through full professor (p<0.001).

Conclusion

The field of vitreoretinal surgery has significantly fewer women compared to men, with women producing fewer publications and having less scholarly impact. H-index and total number of publications are also associated with a higher academic rank. Furthermore, full professors are more likely to be men, while assistant professors are more likely to be women. Future efforts should be aimed at reducing the gender disparity in vitreoretinal surgery.

## Introduction

In the United States (US), women make up approximately 37.1% of all physicians, 25% of all ophthalmologists, and 29.6% of academic ophthalmology faculty [1,2]. The number of women in ophthalmology has been on the rise, and the percentage of women in the field is expected to increase at a faster rate as women now constitute 50% of all US medical graduates and 44.3% of ophthalmology residents, reflecting the trend in other medical fields as well [1]. However, women are still underrepresented in the field of ophthalmology as well as other specialties like urology and orthopedic surgery [3-6]. Furthermore, there are still gender disparities on a national scale among leadership positions [7,8]. Previous studies have found that there are gender inequities in top positions such as editor-in-chief and society presidents [8]. Tuli et al. found that the sex ratios in each academic rank, i.e., associate professor, assistant professor, and full professor, have not changed significantly from 2003 to 2017 [6,8]. It has been suggested that the gender inequity in academic rank might be due to the reluctance of female physicians to continue subspecialty training and their disproportionate entry into primary care and nonsurgical specialties [9,10].

In academic medicine, faculty members are evaluated for academic advancements based on their medical education, clinical performance, and publication productivity, though publication productivity may arguably be the most critical factor for promotion [2,8,11,12]. To measure a physician’s research activity, the number of publications and/or citations that are attributed to their publications, grant recognitions, and awards are evaluated [13]. However, this method is not reliable since it does not provide a comprehensive view of the physician’s publication productivity. To overcome this, the Hirsch index (h-index) has been used to quantify an author’s publication productivity. The h-index is a metric that assesses the entirety of an author’s scholarly output and it provides an estimate of the author’s cumulative impact. The h-index is defined as the number of published articles that have received at least h citations, excluding the articles cited fewer than h times [14]. Thus, the h-index provides a more accurate picture of the quantity and significance of a physician’s work. Previous studies have also shown a strong correlation between academic rank and the h-index in several specialties, including surgery [9,15,16].

While the percentage of women in ophthalmology is lower compared to the percentage of men, the field of vitreoretinal surgery, in particular, has the lowest percentage of women across all subspecialties (e.g., anterior segment, oculoplastics), and it stands at only 19% [17]. There is scarce data about the academic rank and scholarly impact of women ophthalmologists in vitreoretinal surgery. Also, to our knowledge, there has not been an in-depth analysis of academic retina ophthalmologists’ h-index associated with their academic rank and comparison of gender. In light of this, we aimed to investigate gender differences among vitreoretinal specialists in the US with respect to academic rank and publication productivity.

## Materials and methods

This cross-sectional study identified the ophthalmology residency programs in the US that participated in the 2022 San Francisco Match (sfmatch.org). There was a total of 116 ophthalmology residency programs, and none of the programs were excluded.

Official institutional websites were accessed to obtain information regarding gender, scholarly impact or publication number, and academic rank of all academic vitreoretinal faculty. When needed, confirmation of gender was determined through various means, including identification via photographs, examination of pronouns, physician profiles, and additional online resources. Any faculty member whose academic rank could not be determined from the official institutional websites and their online profiles was excluded from this analysis.

The scholarly activity was evaluated by entering the faculty’s first and last name and middle initial when available into the National Library of Medicine PubMed website (https://www.ncbi.nih.gov/pubmed). The total number of peer-reviewed publications was recorded. To confirm the total number of publications, we utilized the Scopus database (Elsevier, https://scopus.com). Furthermore, this database was used to determine the total number of publications that cited the author’s paper as well as their h-index. Covering over 40 million publication records from 22,000 peer-reviewed venues, the Scopus database has been used in previous analyses of the h-index across many medical specialties worldwide [5,15,16,18,19]. To ensure that all publications of each faculty member were captured, we conducted online searches of their curricula vitae and Scopus website profiles for any alternative names that may have been utilized in the past. The h-index, which measures the scholarly productivity and the impact of an author, was calculated based on the highest number of publications an author has been credited for with at least the same number of citations [14]. The citation search was conducted from January 2023 to March 2023.

Data were recorded and analyzed using Microsoft Excel 2016 (Microsoft Inc, Redmond, WA). For statistical analysis and comparison, a binary logistic regression model was performed for genderwise comparison in terms of scholarly productivity, publication quantity, and academic rank of the retina specialists. Additionally, Wilcoxon rank-sum tests were conducted to compare academic productivity metrics, including the medians of the h-index, between genders. Descriptive statistics were analyzed for the median, mean, range, and standard error of the mean (SEM) data. The statistical significance threshold was set at p<0.05, and these comparisons were performed using IBM SPSS version 18 statistical software (IBM Corp., Armonk, NY).

## Results

A total of 467 academic vitreoretinal specialists were identified from the 116 academic ophthalmology residency programs in the US. Among them, 345 (73.9%) were men while 122 (26.1%) were women (p<0.001). Furthermore, our analysis showed that there was a significant difference between the average number of publications for men and women (p<0.001). Women had an average of 52.5 ± 5.1 publications, while men had an average of 90.5.

Gender differences in academic rank and publication productivity

**Figure 1:**
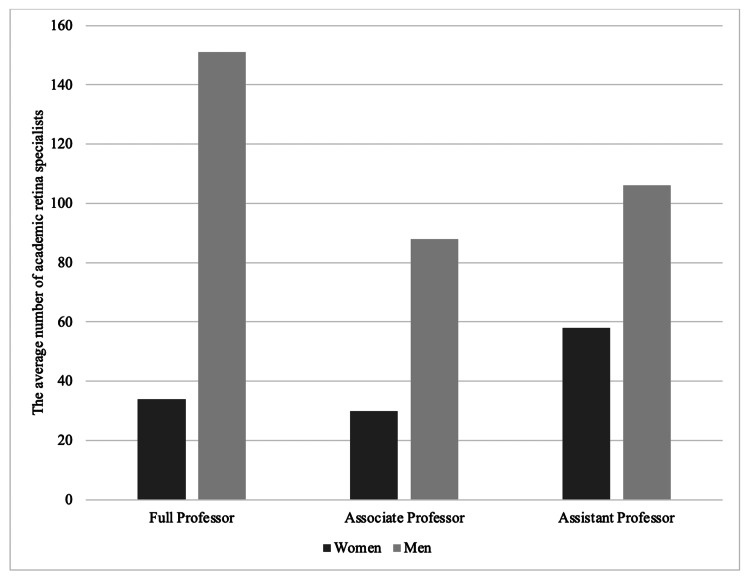
Gender differences in academic rank (full professor, associate professor, and assistant professor) among academic retina specialists in the United States

**Figure 2:**
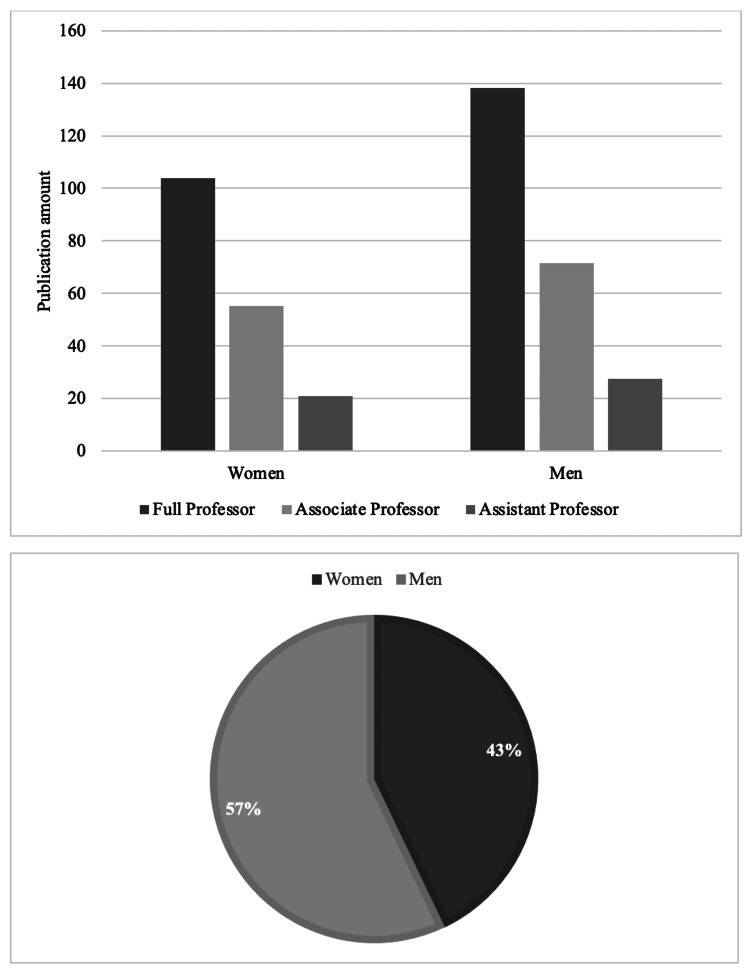
The column graph: gender differences in publications by academic rank among academic retina specialists in the United States. The pie chart: the percentage of publications by gender

When the academic ranks were analyzed, such as associate professor, assistant professor, and full professor of ophthalmology, there was a total of 164 assistant professors in vitreoretinal surgery (Figure 1). Among them, 106 (64.6%) were men while 58 (35.4%) were women. Regarding the mean number of publications among assistant professors, men had a higher number of publications (27.6 ± 3.9) while women had an average of 20.9 ± 2.9 publications (p<0.001) (Figure 2). As for associate professors, there was a total of 118 academic vitreoretinal specialists, which included 88 (74.6%) men and 30 (25.4%) women (Figure 1). Regarding the mean number of publications among associate professors, there was a significant difference (p<0.001) by gender, with a mean publication number of 71.6 ± 7.7 for men, and 55.3 ± 8.9 for women (Figure 2). As for full professors, a total of 185 academic vitreoretinal specialists were identified, with 151 (81.7%) men and 34 (18.3%) women (p<0.001) (Figure 1). Among them, men vitreoretinal surgeons had 138.1 ± 10.3 publications on average while women vitreoretinal surgeons had 103.7 ± 11.9 publications on average (p<0.001) (Figure 2). 

Gender differences in publication productivity based on h-index

Publication productivity in terms of the h-index was evaluated among vitreoretinal specialists based on gender. A higher h-index was correlated with a higher academic rank from assistant professor through full professor (p<0.001). The mean h-index was 7.4 ± 0.7 for assistant professors, 15.2 ± 1.2 for associate professors, and 28.1 ± 1.3 for full professors. Men had a higher publication productivity or scholarly impact (15.2 ± 0.82) compared to women (h-index=12.8 ± 0.99) (p=0.0004). When the median numbers were analyzed, women also had a lower median number (h-index=10) compared to men (h-index=15).

**Figure 3:**
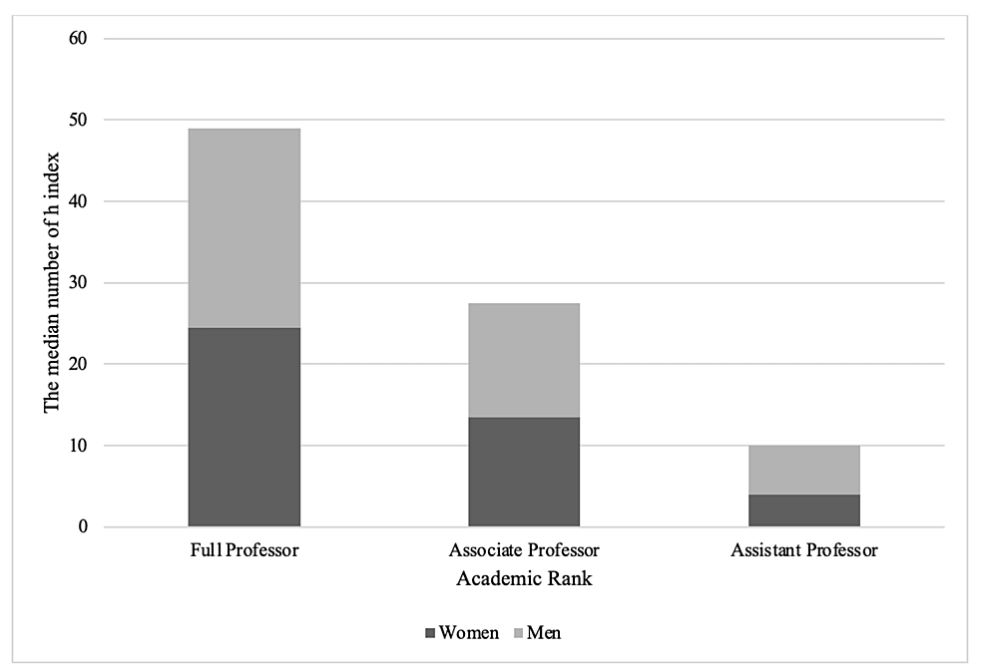
Gender differences in terms of the median number of h-index by academic rank among academic retina specialists in the United States

When analyzed by the academic rank, the h-indices showed no significant difference between women and men for associate and full professors (p=0.30 and p=0.42, respectively), while there was a significant difference among assistant professors (p=0.03), as shown in Figure 3.

## Discussion

Currently, the number of women graduating from medical school is equivalent to or slightly higher than that of men, though women remain underrepresented in the field of ophthalmology [6,17,20]. According to the American Medical Association (AMA), only 22.7% of ophthalmologists involved in direct patient care are women, and 90% of current ophthalmology chairs are men, highlighting a significant gender gap in leadership positions [20]. Several previous studies have raised concerns about the underrepresentation of females in ophthalmology [11,21]. Unfortunately, this issue is not limited to ophthalmology, as other specialties such as orthopedic surgery, urology, plastic surgery, and otolaryngology exhibit similar gender disparities [22,23]. The aim of our study was to investigate the gender disparities regarding publication productivity and academic rank among academic vitreoretinal specialists in the US.

Overall, our study found that there was a significantly smaller proportion of women compared to men in the subspeciality of vitreoretinal surgery. Furthermore, in each academic rank category, we noted a significant underrepresentation of women (p<0.001). While a higher number of men hold the rank of full professor, a higher number of women hold the rank of assistant professor in the retina specialty. Our findings are consistent with previous studies that have shown that female faculty are disproportionately underrepresented in leadership positions within ophthalmology [2,11,24]. Another study by Lopez et al. also found a higher proportion of men serving in senior faculty positions than women [11].

Thus, the gender gap in academic ophthalmology and in vitreoretinal surgery still persists, with fewer women in leadership roles. When gender disparities in academic ranks were evaluated in our study in terms of the number of publications, a disproportionally higher number of publications were by men in each academic rank when compared to women. This might be due to women facing greater challenges in balancing their personal life, which includes taking care of their family, loved ones, and performing household tasks, and professional lives, resulting in a less extensive pursuit of promotions or leadership roles compared to men [25]. A recent study on academic cornea specialists and pediatric ophthalmologists revealed that women were less productive in terms of publications than men [2,26]. Several factors may contribute to this finding, including the fact that women ophthalmologists face biases in the medical field that discourage them from engaging in and publishing research [25,27]. Also, one of the factors can be the uneven distribution of research funding between women and men. Women are given less research funding compared to men [28]. It has been shown that when evaluating scientific merit, peer reviewers of research grants tend to overestimate male accomplishments and underestimate female performance, indicating a gender bias in the review process [29]. Another factor can be the gender disparities in mentorship. Women receive less mentorship than men, and this lack of mentorship could negatively affect women’s career development and achievements [30,31]. One study showed that women who receive mentorship produce more publications and engage in more research activity than those who do not receive mentorship [31].

Furthermore, it is crucial to assess gender disparities in relation to scholarly impact or publication productivity. The h-index serves as a vital tool to evaluate an individual’s publication output and citation impact. Previous research has demonstrated that the h-index tends to increase with higher academic rank and observed a significantly lower h-index among female compared to male physicians in varying specialties [32-35]. Our study not only confirms these findings but also takes a step further by analyzing the h-index in the context of a specific ophthalmology subspecialty, the retina subspecialty. The retina subspecialty has the lowest percentage of women in the field of ophthalmology, and our study is the first to examine the gender difference in h-index scores within the subspecialty.

While the h-index is used worldwide for measuring a physician’s publication productivity, it has limitations and has received criticism from several corners, which needs to be considered. It is important to note that a single metric can only provide a rough approximation of an individual’s overall profile [33,36]. Additionally, if a full professor has not published much over many years, their h-index may still remain the same as that of a younger professor who has published a few highly cited articles. It is also worth considering that if one’s name appears on a paper, their h-index automatically increases, without taking into account their authorship position [37]. To address these limitations, future studies could explore modified h-indices that factor in authorship position. Furthermore, the h-index does not account for journal quality, although highly cited publications tend to come from higher-quality journals.

There are several limitations to our study that must be taken into account. One significant limitation was our reliance on the online availability of information. We collected data from institutional websites, and we were not able to directly confirm the accuracy of the information with each department. Secondly, our study used the National Library of Medicine PubMed and Scopus databases to obtain data on the publication activity of each faculty. However, it is possible that some alternative names were not identified, limiting the accuracy of our searches. Alternative names, such as a maiden name, were challenging to locate and this may have affected our analysis of scholarly activity. Furthermore, gender was assigned based on the evaluation of photographs, pronouns, and names, and it is possible that the assigned gender might not align with how some of these individuals report their gender. Lastly, our study was limited to academic vitreoretinal specialty only, and it did not look into their counterparts in private practice, which may have unintentionally excluded women vitreoretinal specialists who conduct and publish research in the private setting.

## Conclusions

The vitreoretinal subspecialty has significantly fewer women compared to men. Men have a higher number of publications with a higher h-index and occupy higher academic positions as compared to their female counterparts. H-index and total number of publications are also associated with a higher academic rank. Furthermore, full professors are more likely to be men, while assistant professors are more likely to be women. Future efforts should be aimed at reducing the gender disparity in vitreoretinal surgery. Further studies should be conducted to evaluate the factors that contribute to these disparities as well as the changes that can be implemented to narrow the gender gap regarding scholarly impact in the field of ophthalmology.
